# Functional cure for chronic hepatitis B on hepatocellular carcinoma prevention: Evidence and clinical implications

**DOI:** 10.1016/j.iliver.2026.100235

**Published:** 2026-04-28

**Authors:** Wen Kang, Yu-Shen Liu, Tian-Ping Wang, Shu-Ming Zhang, Yu Li, Ye Zhang

**Affiliations:** a Department of Infectious Diseases, Tangdu Hospital, Fourth Military Medical University, Xi'an 710038, Shaanxi, China; b School of Basic Medicine and Clinical Pharmacy, China Pharmaceutical University, Nanjing 211198, Jiangsu, China; c Clinical Pharmacy Programme, The University of Manchester, Manchester M13 9PL, United Kingdom; d Department of Infectious Diseases, Shaanxi Provincial People's Hospital, Xi'an 710068, Shaanxi, China

**Keywords:** Chronic hepatitis B, Functional cure, Hepatocellular carcinoma, Antiviral therapy, Pegylated interferon-α, Nucleos(t)ide analogs

## Abstract

Chronic hepatitis B virus (HBV) infection is a leading cause of hepatocellular carcinoma (HCC). The functional cure for chronic hepatitis B (CHB), defined as sustained hepatitis B surface antigen (HBsAg) seroclearance for at least 24 weeks, undetectable hepatitis B e antigen and HBV DNA, and normalized liver functions, has emerged as a pivotal therapeutic goal due to its association with a significant reduction in long-term complications, particularly HCC. This review comprehensively summarizes the epidemiological burden of HBV-related HCC, elucidates the mechanisms underlying HBV-induced hepatocarcinogenesis, and identifies the key risk factors for HCC development in CHB patients, *e*.*g*., advanced age, cirrhosis, family history of HCC, and high HBV DNA levels. A critical focus of the review is the efficacy of first-line antiviral therapies in achieving functional cure and preventing HCC. Nucleos(t)ide analogs effectively inhibit HBV replication and reduce HCC risk by approximately 50%, but they rarely achieve HBsAg seroclearance, leaving a persistent HCC risk with 5-year cumulative incidence ≥7%. Pegylated interferon-α not only achieves higher HBsAg seroclearance rate but also reduces HCC risk by nearly 90%, with a 5-year cumulative incidence < 3%. Long-term follow-up studies confirm that HBsAg seroclearance sustains low HCC risk with 5-year cumulative incidence < 2% even in high-risk CHB patients. Additionally, this review discusses clinical strategies to optimize CHB management for HCC prevention, emphasizing the priority of pursuing functional cure and the necessity of long-term HCC surveillance. Collectively, this review synthesizes evidence demonstrating that achieving functional cure, particularly through pegylated interferon-α-based strategies, should be a primary treatment goal to maximally reduce the long-term risk of HCC in CHB patients.

## 1 Introduction

Hepatitis B virus (HBV) infection remains a major global public health burden, with an estimated 257 million chronic carriers worldwide.^1^ Among the severe complications of chronic HBV infection, hepatocellular carcinoma (HCC) is the third leading cause of cancer-related death globally.^2^ The epidemiological burden of HBV-related HCC is disproportionately concentrated in China. In 2022, China accounted for 368,000 new HCC cases (42.5% of the global total) and 317,000 HCC deaths (41.8% of the global total),^2^^,^^3^ with up to 85% of these cases attributed to HBV infection.^4^ Projections using an individual-based Markov model indicate that the annual incidence of HBV-related HCC in China rose from 235,000 in 2006 to 326,000 by 2023, driven by population aging and the long natural history of chronic HBV infection.^5^

Persistent HBV infection progresses through distinct clinical phases, including hepatitis B e antigen (HBeAg)-positive and HBeAg-negative chronic hepatitis, which may advance to liver cirrhosis. The risk of HCC increases exponentially with disease progression.^6^ The lifetime risk of liver cirrhosis and/or HCC in chronic HBV infection ranges from 15% to 40%, and the relative risk of HCC in chronic hepatitis B (CHB) patients is 14 to 233 times higher than that in the general population 7, 8, 9. These statistics underscore the urgent need for effective strategies to prevent HBV-related HCC. Antiviral therapy is the cornerstone of CHB management, with two main first-line treatment options: oral nucleos(t)ide analogs (NAs) (including entecavir [ETV], tenofovir disoproxil fumarate [TDF], tenofovir alafenamide [TAF], and tenofovir aminufenamide) and pegylated interferon-α (PEG-IFN-α) subcutaneous injection.^10^ NAs effectively suppress HBV DNA replication^11^ but rarely achieve HBsAg seroclearance, leaving a residual HCC risk.^12^ In contrast, PEG-IFN-α exerts both antiviral and immunomodulatory effects, enabling a subset of patients to achieve functional cure. The functional cure of CHB is defined as sustained hepatitis B surface antigen (HBsAg) seroclearance (≥24 weeks post-therapy), undetectable HBeAg and serum HBV DNA, as well as normalized liver function following a finite course of therapy.^13^ Functional cure of CHB is strongly correlated with maximal reduction in HCC risk.^14^

This review aims to synthesize the evidence on the impact of CHB functional cure on the development and risk of HBV-related HCC. We first outline the epidemiology of HBV-related HCC, then discuss the mechanisms of HBV-induced hepatocarcinogenesis and key risk factors for HCC development in CHB patients. We subsequently investigate the HCC-preventive effects of NAs and PEG-IFN-α, with a focus on the clinical benefits of HBsAg seroclearance. Finally, we provide clinical recommendations to optimize CHB management for HCC prevention, highlighting the critical role of actively pursuing functional cure.

## 2 Epidemiology of HBV-related HCC

Nearly 50% of global HCC cases are associated with HBV infection, with significant geographical variation in incidence.^15^^,^^16^ In China, the high burden stems from a large population of chronic HBV carriers and historical delays in the implementation of widespread vaccination programs. Comprehensive prevention strategies (*e*.*g*., universal infant hepatitis B vaccination with emphasis on timely birth-dose and 3-dose coverage, dramatically reduced the mother-to-infant transmission and early childhood acquisition of HBV) lead to estimated HBsAg prevalence of 5.6% in the general population and 0.1% in children aged < 5 years in 2022.^17^ However, China still accounts for 69% of global HBV-related HCC cases.^18^ This is partly due to a projected increase in incidence driven by the aging of patients infected before the national vaccination program was launched in 1992.^18^ In other East Asian countries, such as Japan and Republic of Korea, the 5-year cumulative HBV-related HCC incidence is higher (> 10%) in untreated patients compared with NAs-treated CHB patients 19, 20, 21, 22. In the United States, 31,671 HCC patients were newly diagnosed in California, with 3271 (10.3%) due to HBV infection. The incidence rate of HBV-related HCC per 100,000 of the population remained high in Asian and Pacific Islander subgroups, though the overall rate has declined remarkably.^23^

The incidence and mortality of HBV-related HCC is shaped by HBV vaccination coverage, antiviral therapy access, and population demographics. In China, annual deaths of HBV-related HCC decreased by 23.34% from 1990 to 2019, with an age-standardized mortality rate (ASMR) reduction of 5.17% per year (95% confidence interval (CI): −6.00 to −4.33) due to the widespread vaccination.^24^ However, projections indicate a continuous rise in HBV-related HCC incidence until 2030, as chronic HBV-infected patients in the pre-vaccination era (1970s–1980s) reach the high-risk age (> 40 years).^18^ In the United States, a cross-sectional study conducted on 188,280 HCC-related deaths between 2006 and 2022 found that the annual percentage change in ASMR was 4.1% from 2006 to 2009, and declined to 1.8% from 2009 to 2022. While ASMRs increased for alcohol-associated liver disease and metabolic dysfunction-associated steatotic liver disease (MASLD), they decreased for viral hepatitis-related mortality, which were mainly due to the improved vaccination coverage and antiviral therapy access.^25^ These trends highlight the potential of preventive strategies (vaccination and antiviral therapy) to reduce HBV-related HCC burden over time.

## 3 Mechanisms of HBV-induced hepatocarcinogenesis

### 3.1 Direct viral effects

#### 3.1.1 HBV DNA integration

HBV DNA integration into the host genome is a hallmark of chronic HBV infection and a key driver of hepatocarcinogenesis. During replication, HBV relaxed circular DNA (rcDNA) is converted to covalently closed circular DNA (cccDNA) in the nucleus, but a subset of rcDNA randomly integrates into host chromosomes, an early step in clonal tumor expansion. Integration preferentially occurs at fragile sites or cell cycle-regulated genes (*e*.*g*., telomerase reverse transcriptase [*TERT*], myelocytomatosis viral oncogene homolog [*MYC*]).^26^^,^^27^ HBV DNA integration extensively reshapes genomic structure, activates oncogenes, inactivates tumor suppressor genes, induces genomic instability, and directly promotes insertional mutagenesis of various cancer-related genes.^28^ A study has identified distinct patterns and characteristics of HBV DNA integration in HBV-related HCC. Eighty-seven integration sites were determined in 20 plasma samples from HBV-related HCC patients, with significant enrichment in intronic regions (53%, 46/87) and integration sites were particularly targeted in cancer-related pathways.^29^ Another study has shown that HBV DNA integrations are common in chromosomes 5, 8, 10, and 19 in HCC tissue, whereas chromosomes 1 and 2 are frequent integration sites in non-tumor liver tissue.^30^ HBV integration-targeted genes (ITGs) are enriched in several cancer-related pathways, including mitogen-activated protein kinases, extracellular matrix-receptor interaction, and the hedgehog signaling pathway. HBV-related HCC patients with a high number of HBV DNA integrations are generally 40–60 years old and exhibited shorter disease-free and overall survival periods than those without integrations in certain ITGs.^31^ For example, HBV-related HCC patients with HBV DNA integration into the *TERT* promoter had a higher male-to-female ratio, higher cirrhosis rate, and increased risk of early recurrence and mortality after resection.^26^^,^^32^^,^^33^

Notably, there is a stepwise reduction in integration events from HBeAg-positive to HBeAg-negative and HBsAg-negative patients.^34^ The levels of HBV DNA integration are reduced by 12.25-fold at 78 weeks post-NAs therapy compared with baseline.^35^ CHB patients receiving or having received antiviral therapy have a significantly lower percentage of ITGs and fewer chimeric reads than treatment-naïve patients.^36^ Spatial transcriptomics has revealed a low extent of transcriptionally active HBV DNA integration in CHB patients with HBsAg seroclearance.^36^ However, integrated HBV DNA and cccDNA maintained transcriptional activity in intrahepatic HBsAg-positive CHB patients who achieved functional cure with PEG-IFN-α therapy.^37^ Thus, long-term anti-HBV therapy may reduce but not eliminate HBV DNA integration,^35^ which can still accelerate HCC development.^38^

#### 3.1.2 Expression of viral proteins

HBV encodes several oncogenic proteins, with HBsAg and hepatitis B x protein (HBx) being the most well-characterized. HBsAg promotes proliferation and tumorigenicity of HBV-positive HCC cells,^39^ mainly through interaction with β2-glycoprotein Ⅰ to activate Toll-like receptor 4/myeloid differentiation factor 88/IκBα axis,^40^ induction of oncogenic long noncoding RNAs *via* the nuclear factor-κB (NF-κB) pathway,^41^ and promotion of stemness of HCC.^42^ HBx is a multifunctional regulatory protein that drives viral replication and modulates HCC initiation, progression, invasion, and metastasis.^43^ The key cellular and molecular mechanisms regulated by HBx that induce hallmarks of HCC include sustaining proliferative signaling, evading growth suppressors, avoiding immune destruction, facilitating replicative immortality, aiding in tumor-promoting inflammation, triggering invasion and metastasis, prompting angiogenesis, inducing genome instability, resisting cell death, regulating cellular metabolism, and inducing cancer stem cell-like properties.^43^

### 3.2 Indirect host-mediated processes

#### 3.2.1 Chronic liver inflammation and fibrosis

Chronic liver inflammation is a central driver of HBV-related HCC. Persistent HBV replication causes hepatocyte necrosis, triggering an inflammatory response characterized by recruitment of T cells, macrophages, and neutrophils into the liver.^44^ These immune cells release pro-inflammatory cytokines and chemokines that activate the NF-κB signaling pathway, promoting cell proliferation and angiogenesis.^41^^,^^45^ Chronic liver inflammation leads to fibrosis, which is both reversible and progressive. As fibrosis advances to cirrhosis, the formation of regenerative nodules is predisposed to malignant transformation due to increased oxidative stress, genomic instability, epigenetic modifications, gut microbiota dysbiosis, and immune escape, and dysregulated signaling pathways. This highlights the complex interactions and synergies among HBV, fibrosis, cirrhosis, and HCC.^46^

#### 3.2.2 Immune dysregulation

Chronic HBV infection is characterized by an impaired viral-specific immune response, which permits persistent viral replication and chronic inflammation. HBV infection modifies the composition and functional state of immune cells, resulting in immune cell dysfunction or exhaustion within the HCC tumor microenvironment.^47^ HCC tissues from HBV-positive and HBV-negative cases demonstrated that the proportions of T cells, B cells, and natural killer (NK) cells were remarkably elevated in HBV-infected livers, suggesting increased infiltration or proliferation of these cell types.^47^ Intrahepatic T cells from HCC tissues express higher levels of exhaustion markers and show higher clonal expansion of CD4^+^ regulatory T cells (Tregs), along with increased cccDNA and pregenomic RNA (pgRNA) levels, indicating heterogeneity in T cell exhaustion in the tumor microenvironment of HBV-related HCC.^48^ The enrichment of HBV-specific PD-1^+^CD8^+^ tissue-resident memory T cells in the tumor borders of HBV-related HCC patients is associated with hepatic damage, fibrosis, a higher *TP53* gene mutation rate, and more pronounced HBV integration.^49^

## 4 Risk factors for HBV-related HCC

Key risk factors for HBV-related HCC are summarized in Table 1 as follows and can be categorized into demographic, viral, host, and lifestyle-related factors. Among these, advanced age (> 40 years), male sex, high HBV DNA levels, presence of cirrhosis, and HCC family history are consistently the strongest predictors. For example, detectable HBV DNA levels during NAs treatment is one of the independent predictive factors of HCC risk.^50^ A large-scale non-cirrhotic CHB cohort revealed that baseline moderate HBV viral load (10^6^ IU/mL) confers the highest HCC risk.^51^ Similarly, baseline viral load was significantly associated with HCC risk in non-cirrhotic HBeAg-positive CHB patients despite antiviral treatment.^52^ Furthermore, untreated HBV-related compensated cirrhotic patients have an annual HCC incidence of 2.03%–3.37%, compared to 0.12%–0.49% in untreated non-cirrhotic CHB patients.^9^^,^^53^ Family history of HCC was associated with an odds ratio of 3.58 for HCC development in CHB patients (95% CI: 2.53 to 5.06),^54^ and is also linked to reduced overall survival (hazard ratio [HR]: 1.574, 95% CI: 1.171 to 2.116) and recurrence-free survival (HR: 1.534, 95% CI: 1.176 to 2.002) among patients undergoing curative liver resection for HBV-related HCC.^55^ Importantly, many of these factors, such as the high viral load and active liver inflammation, are directly mitigated by successful antiviral therapy leading to functional cure.

**Table 1:** Key risk factors for HBV-related HCC in CHB patients.

Category	Risk factor	Potential mechanisms	References
Demographic factors	Age > 40 years	Genomic instability, gene expression changes, and DNA methylation	^56^
	Male gender	Androgen receptor facilitated HCC cell growth.	^57^
		Males expressed higher levels of aflatoxin metabolism-related genes.	^58^
Viral factors	Persistent high HBV DNA	Increased HBV integration, inflammation, and viral protein expression	^59^
	HBeAg positivity	Marker of active replication and association with severe inflammation	^60^
	HBV genotype C	Higher replication efficiency and severe liver disease progression	^61^
Host factors	Cirrhosis	Regenerative nodules, oxidative stress, and genomic instability	^62^
	Family history of HCC	Genetic polymorphisms and shared environmental exposures	^63^^,^^64^
	Co-infection with HCV, HDV or HIV-1	Accelerated inflammation and fibrosis progression	^65^
Lifestyle factors	Alcohol consumption	Activation of transcription factor 4/lysosomal phospholipase A2-mediated bis(monoacylglycero)phosphate metabolism	^66^
	Smoking	Carcinogens-induced DNA damage and gene mutation Triggering HSCs activation through pro-inflammatory cytokines	^67^
	Diabetes mellitus	Insulin resistance promoted the release of pro-inflammatory cytokines	^68^
	Aflatoxin exposure	Induction of p53 mutation, and synergization with HBV infection	^69^

Abbreviations: HCC, hepatocellular carcinoma; HBV, hepatitis B virus; HBAg, hepatitis B e antigen; HCV, hepatitis C virus; HDV, hepatitis D virus; HIV-1, human immunodeficiency virus-1; HSCs, hepatic stellate cells.

## 5 Effectiveness of antiviral therapies in preventing HBV-related HCC

As summarized in Table 2^21^^,^^22^^,^^70^, ^71^, ^72^, ^73^, ^74^, ^75^, ^76^, ^77^, ^78^, ^79^, ^80^, ^81^, ^82^, ^83^, ^84^, ^85^, while NAs therapy significantly reduces HCC risk compared with no treatment, the residual risk remains substantial (5-year cumulative incidence typically ≥7%).^21^^,^^22^^,^^70^, ^71^, ^72^, ^73^, ^74^, ^75^ In contrast, IFN-α/PEG-IFN-α-based regimens are associated with a markedly lower HCC incidence, with most studies reporting 5-year rates < 3%.^76^, ^77^, ^78^, ^79^, ^80^, ^81^, ^82^, ^83^, ^84^, ^85^

**Table 2:** The cumulative HCC incidence in different CHB cohorts with NAs or IFN-α/PEG-IFN-α-based therapy.

Characteristics of CHB	Countries and territories	Cases (*n*)	Therapeutic strategies	Observation period	HCC incidence(%)	References
Treatment-naïve	Republic of Korea	1336	ETV (*n* = 671)	4.4 years (range: 1.0–7.4 years)	7.3 (5-year)	21
			TDF (*n* = 665)		6.3 (5-year)	21
Treatment-naïve	Republic of Korea	2897	ETV (*n* = 1484)	5 years	9.3 (5-year)	22
			TDF (*n* = 1413)		7.7 (5-year)	22
Treatment-naïve, HBV DNA > 2000 IU/mL	Taiwan, China	1397	ETV	10 years	4.0 (3-year)	70
					9.1 (5-year)	70
					15.8 (10-year)	70
HBV DNA > 2000 IU/mL, ALT ≥2 × ULN	Taiwan, China	21,595	ETV, lamivudine, telbivudine	3.46 years	7.32 (7-year)	71
Treatment-naïve, ALT ≥2 × ULN, HBeAg +: HBV DNA ≥20000 IU/mL HBeAg –: HBV DNA ≥2000 IU/mL	Republic of Korea	1378	ETV, lamivuidne, clevudine	incomplete response: 4.31 years;	18.8 (5-year)	72
				complete response: 3.13 years	11.4 (5-year)	72
Treatment-naïve	China, Japan, Republic of Korea, United States	5537	ETV (*n* = 4837)	5 years	7.25 (5-year)	73
			TDF (*n* = 700)	3.23 years	3.19 (5-year)	73
Treatment-naïve	Japan	905	ETV, TDF, TAF	6.2 years (range: 1.0–15.7 years)	3.09 (3-year)	74
					6.48 (5-year)	74
					10.28 (10-year)	74
Starting NAs therapy based on HBV DNA, ALT, and histological progression in different natural history	Japan	445	ETV, TDF, TAF	7.4 years	10.3(total)	75
HBeAg +, male	Taiwan, China	67	IFN-α	7.4 years (range: 1.1–11.5 years)	1.5 (total)	76
HBeAg –, ALT ≥2 × ULN	Greece	209	IFN-α	6 years (range: 1–13.5 years)	8.1 (total)	77
HBeAg + with active hepatitis	Taiwan, China	233	IFN-α-natural, IFN-α-2a, IFN-α-2b	1.1–16.6 years	2.1 (15-year)	78
Treatment-naïve	Republic of Korea	641	IFN-α-2b	9.42 years	0.4 (5-year)	79
					3.2 (10-year)	79
CHB patients underwent liver biopsy	Taiwan, China	153	PEG-IFN-α-2a mono- or combined with NAs	up to 5 years	0.7 (total)	80
HBeAg + without cirrhosis	Chongqing, China	183	PEG-IFN-α-2a	up to 5 years	0 (total)	81
ALT ≥2 × ULN, HBeAg +: HBV DNA ≥20000 IU/mL HBeAg –: HBV DNA ≥2000 IU/mL	Shanghai, China	430	IFN-α or PEG-IFN-α-2a/2b mono- or combined with NAs	5.41 years (interquartile range: 2.56–7.91 years)	2.7 (total)	82
CHB patients	Beijing, China	465	IFN-α combined with ETV	up to 9 years	2.2 (total)	83
					6.7 (9-year)	83
CHB patients underwent liver biopsy	Xiamen, China	877	IFN-α or PEG-IFN-α-2a/2b mono- or combined with NAs	5.2 years (interquartile range: 4.5–6.0 years)	0.6 (total)	84
NAs pretreatment ≥24 weeks, undetectable HBV DNA, intermediate to high risk of HCC development (PARADISE study)	Shanghai and other 6 provinces in China	128	PEG-IFN-α-2b combined with NAs therapy	up to 5 years	0 (2-year)	85

Abbreviations: HCC, hepatocellular carcinoma; CHB, chronic hepatitis B; HBV, hepatitis B virus; ALT, alanine aminotransferase; HBeAg, hepatitis B e antigen; ULN, upper limit of normal; NAs, nucleos(t)ide analogs; IFN-α, interferon-α; PEG-IFN-α, pegylated interferon-α; ETV, entecavir; TDF, tenofovir disoproxil fumarate; TAF, tenofovir alafenamide.

### 5.1 NAs therapy

NAs are oral, well-tolerated antiviral agents that suppress HBV DNA polymerase, achieving virological response in over 90% of CHB patients within 1 year. NAs effectively inhibit HBV replication and reduce HCC risk by approximately 50%, but they rarely achieve HBsAg seroclearance, leaving a persistent HCC risk with 5-year cumulative incidence often exceeding 7% (Table 2). The residual HCC risk in NAs-treated CHB patients is attributed to persistent cccDNA, integrated HBV DNA, and ongoing low-grade liver inflammation. Chow et al.^86^ found that HBV integration remained detectable even 10 years post NAs therapy, with a median 0.93 log reduction in integration frequency and a 1.02 log reduction in hepatocyte clone size compared with baseline. It is crucial to emphasize that despite this residual HCC risk, NAs remain the cornerstone of antiviral therapy for the vast majority of CHB patients globally. They are essential for achieving potent and sustained viral suppression, halting or reversing liver fibrosis, preventing clinical decompensation, and significantly reducing the risk of HCC. Their excellent oral tolerability and high genetic barrier to resistance make them a practical and foundational option for long-term management, particularly for patients clinically ineligible for or non-responsive to IFN-based therapies.

### 5.2 IFN-α/PEG-IFN-α-based therapy

IFN-α/PEG-IFN-α is administrated subcutaneously and exerts dual antiviral and immunoregulatory effects. It inhibits HBV replication by inducing antiviral genes, and enhances clearance of HBV-infected hepatocytes by activating the viral-specific immune responses.^13^ PEG-IFN-α-2b combined with TDF achieves sustained functional cure in CHB patients, with cumulative functional cure rate of 31.4% after 2 years of therapy.^87^ Critically, IFN-α/PEG-IFN-α-based therapy was associated with a far greater reduction in HCC risk (nearly 90%) compared with untreated or NAs-treated CHB patients, with most 5-year cumulative incidence < 3% (Table 2). PEG-IFN-α not only effectively inhibited the transcriptional activity of integrated HBV DNA and cccDNA^37^ but also enhanced the NK cell functionality and specific T cell responses in CHB patients.^88^ The levels of CXCL10, CD8, CD19, mature B cells, and IFN-γ-secreting CD4^+^ T cells were increased after PEG-IFN-α treatment.^89^ Additionally, PEG-IFN-α-2b combined with sequential low-dose interleukin-2 therapy could restore viral-specific CD8^+^ T cell activity in non-responders to IFN-based treatment.^90^ The immunomodulatory effects are critical for achieving HBsAg seroclearance and long-term reduction in HCC prevention in CHB patients.

## 6 Impact of functional cure or HBsAg seroclearance on HBV-related HCC risk

The definitive clinical evidence for the protective effect of functional cure comes from long-term studies of patients who achieve HBsAg seroclearance, as shown in Table 3^91^, ^92^, ^93^, ^94^, ^95^, ^96^, ^97^, ^98^, ^99^, ^100^. Meta-analyses and large cohort studies consistently demonstrate that the HBsAg loss, whether spontaneous or treatment-induced, is associated with a very low long-term risk of HCC, with 5-year cumulative incidences consistently below 2%.^91^, ^92^, ^93^, ^94^, ^95^, ^96^, ^97^, ^98^, ^99^, ^100^ Despite this profound risk reduction, HCC can still rarely occur, primarily in high-risk individuals such as older males with established cirrhosis at the time of HBsAg seroclearance.^91^^,^^93^^,^^96^ This underscores the need for ongoing HCC surveillance in this subgroup. The protective effects of HBsAg seroclearance against HCC risk mainly mediated by the elimination of cccDNA-containing hepatocytes, the reduction of pool of pre-malignant cells, the normalization of ALT levels, the regression of liver fibrosis, and the restoration of antiviral immunity.

**Table 3:** HCC incidence after HBsAg seroclearance in HBV-infected patients.

Study type	Countries and territories	Cases (*n*)	Time range of patients or literature enrolled	Observation period	HCC incidence(%)	References
Retrospective study	Republic of Korea	829	1997 to 2012	3.2 years (interquartile range: 1.8–6.1 years)	1.6 (5-year)	91
					5.9 (10-year)	91
					15.2 (12-year)	91
Nested case-control study	Alaska, United States	238	1982 to 2013	11.7 years (interquartile range: 6.5–18.3 years)	1.68 (total)	92
Retrospective study	Hong Kong, China	4568	January 2000 to August 2016	3.4 years (interquartile range: 1.5–5.0 years)	0.9 (1-year)	93
					1.3 (3-year)	93
					1.5 (5-year)	93
Retrospective study (ETV/TDF-induced HBsAg loss)	Hong Kong, China	376	January 2005 to December 2016	4.8 years (interquartile range: 2.8–7.0 years)	0.6 (8-year)	94
Retrospective study	Hong Kong, China	7124	January 2000 and March 2019	4.3 years (interquartile range: 2.2–7.6 years)	1.2 (3-year)	95
					1.6 (5-year)	95
					1.9 (7-year)	95
Retrospective study	Hong Kong, China	9769	January 2000 to December 2020	4.6 years (interquartile range: 2.2–8.4 years)	0.9 (5-year)	96
					1.3 (7-year)	96
					2.2 (12-year)	96
Meta-analysis	–	34,952	February 1993 to January 2015	more than 1 year	2.29 (total)	97
Meta-analysis	–	0.19/1000 person-years in 188,316 CHB	1990 to 2018	Not available	0.14/1000 person-years	98
Meta-analysis	–	43,924	January 2000 to January 2020	4.74 years (range: 1.45–12.76 years)	1.88 (total)	99
Meta-analysis	–	63,164	Until May 2021	Not available	0.84 (total)	100

Abbreviations: HCC, hepatocellular carcinoma; HBsAg, hepatitis surface antigen; ETV, entecavir; TDF, tenofovir disoproxil fumarate.

## 7 Clinical strategies to optimize CHB management for HCC prevention

Based on the evidence presented, clinical management of CHB should prioritize the actively pursuing functional cure to maximize HCC prevention, especially in high-risk clinical populations. CHB patients should undergo risk stratification using different validated scoring systems to identify those who require immediate antiviral therapy. High-risk clinical populations mainly include CHB patients with cirrhosis, age > 40 years, family history of HCC, higher HBV DNA levels, or elevated ALT levels. Such patients should be prioritized for therapy aimed at functional cure. PEG-IFN-α is the preferred agent for pursuing functional cure, especially in patients with low baseline HBsAg levels (< 1500 IU/mL), moderate to high ALT level (2 × ULN < ALT < 5 × ULN), HBeAg positivity, and no cirrhosis/compensated cirrhosis. Although some CHB patients do not fully meet the conditions of preferred clinical populations for functional cure,^101^^,^^102^ PEG-IFN-α still can reduce HCC risk for them. First-line NAs should be used for persistent viral suppression in patients with clinical contraindications to PEG-IFN-α (*e*.*g*., decompensated cirrhosis, psychiatric disorders).^10^ CHB patients should be clinically counseled on lifestyle modifications to reduce HCC risk, including avoiding alcohol consumption, smoking cessation, avoiding aflatoxin-contaminated foods, and maintaining a healthy weight to prevent MASLD, which synergizs with HBV to increase the HCC risk. Finally, long-term HCC surveillance is recommended even after HBsAg seroclearance, especially for those with high-risk features for HCC development.

## 8 Conclusion and future directions

Despite significant progress in clinical CHB management, several challenges remain to optimize clinical HCC prevention in CHB patients. First, current PEG-IFN-α-based therapies achieve HBsAg seroclearance in 31.4% of preferred clinical CHB patients. Novel agents under development (*e*.*g*., small interfering RNA, Toll-like receptor agonists, antisense oligonucleotides, and therapeutic vaccines) aim to improve the HBsAg seroclearance and seroconversion rates. Second, new biomarkers (*e*.*g*., HBsAg kinetics, serum cytokines, and gut microbiome) should be identified to predict the therapeutic response to PEG-IFN-α therapy, enabling the development of personalized therapeutic strategies. Third, the safety of discontinuing NAs therapy after HBsAg seroclearance is still not fully elucidated. Large-scale, prospective cohort studies should be performed to evaluate relapse rates and long-term outcomes after discontinuation.

In summary, the functional cure of CHB, which primarily achieved *via* PEG-IFN-α therapy, represents the most effective clinical strategy to reduce HCC risk, with HBsAg seroclearance associated with a 90% reduction in HCC incidence and sustained low risk. Clinical management of CHB should prioritize risk stratification to identify high-risk patients, select therapies aimed at functional cure (especially PEG-IFN-α-based therapy), and implement long-term HCC surveillance even after HBsAg seroclearance. Therefore, in the clinical management of CHB, the pursuing functional cure represents the most effective strategy to alter the natural history of the liver disease and achieve the ultimate goal of preventing HBV-related HCC.

## CRediT authorship contribution statement

**Wen Kang:** Writing – original draft, Methodology. **Yu-Shen Liu:** Writing – review & editing, Methodology. **Tian-Ping Wang:** Writing – review & editing, Methodology. **Shu-Ming Zhang:** Writing – original draft, Methodology. **Yu Li:** Writing – review & editing, Supervision, Investigation, Data curation, Conceptualization. **Ye Zhang:** Writing – original draft, Supervision, Investigation, Data curation, Conceptualization.

## Informed consent

Not Applicable.

## Organ donation

Not Applicable.

## Ethics statement

Not Applicable.

## Animal treatment

Not Applicable.

## Data availability statement

No new data were created or analyzed in this study. Data sharing does not apply to this article.

## Declaration of generative AI and AI-assisted technologies in the writing process

During the preparation of this work the authors did not use generative AI or AI-assisted technologies.

## Funding

None.

## Declaration of interests

The authors declare that they have no known competing financial interests or personal relationships that could have appeared to influence the work reported in this paper.

## References

[1] Stasi C., Silvestri C., Voller F. (2020). Hepatitis B vaccination and immunotherapies: an update. Clin Exp Vaccine Res.

[2] Bray F., Laversanne M., Sung H. (2024). Global cancer statistics 2022: GLOBOCAN estimates of incidence and mortality worldwide for 36 cancers in 185 countries. CA Cancer J Clin.

[3] Xia C.F., Dong X.S., Li H. (2022). Cancer statistics in China and United States, 2022: profiles, trends, and determinants. Chin Med J.

[4] Chan S.L., Wong V.W., Qin S.K. (2016). Infection and cancer: the case of hepatitis B. J Clin Oncol.

[5] Xie J.Z., Wang X., Wang X.R. (2024). Assessing the impact of comorbid type 2 diabetes mellitus on the disease burden of chronic hepatitis B virus infection and its complications in China from 2006 to 2030: a modeling study. Glob Health Res Pol.

[6] Wursthorn K., Manns M.P., Wedemeyer H. (2008). Natural history: the importance of viral load, liver damage and HCC. Best Pract Res Clin Gastroenterol.

[7] Mak L.Y., Cruz-Ramón V., Chinchilla-López P. (2018). Global epidemiology, prevention, and management of hepatocellular carcinoma. Am Soc Clin Oncol Educ Book.

[8] Shi J., Zhu L., Liu S. (2005). A meta-analysis of case-control studies on the combined effect of hepatitis B and C virus infections in causing hepatocellular carcinoma in China. Br J Cancer.

[9] Lin C.L., Kao J.H. (2023). Development of hepatocellular carcinoma in treated and untreated patients with chronic hepatitis B virus infection. Clin Mol Hepatol.

[10] You H., Wang F., Li T. (2023). Guidelines for the prevention and treatment of chronic hepatitis B (version 2022). J Clin Transl Hepatol.

[11] Li F.Y., Zhang Y.D., Liu C. (2024). Clinical outcomes of cessation of nucleoside/nucleotide analogues in Chinese patients with HBeAg-negative chronic hepatitis. B iLIVER.

[12] Broquetas T., Carrión J.A. (2022). Current perspectives on nucleos(t)ide analogue therapy for the long-term treatment of hepatitis B virus. Hepat Med.

[13] Zhang Y., Li Y., Lian J.Q. (2025). Pegylated interferon-α-induced functional cure for special populations with chronic hepatitis B virus infection: current trends, challenges and prospection. Drug Des Dev Ther.

[14] Ren H., Huang Y. (2019). Effects of pegylated interferon-α based therapies on functional cure and the risk of hepatocellular carcinoma development in patients with chronic hepatitis B. J Viral Hepat.

[15] Lee C.M., Lu S.N., Changchien C.S. (1999). Age, gender, and local geographic variations of viral etiology of hepatocellular carcinoma in a hyperendemic area for hepatitis B virus infection. Cancer.

[16] Sayiner M., Golabi P., Younossi Z.M. (2019). Disease burden of hepatocellular carcinoma: a global perspective. Dig Dis Sci.

[17] Shan S., Zhao X.Y., Jia J.D. (2024). Comprehensive approach to controlling chronic hepatitis B in China. Clin Mol Hepatol.

[18] de Martel C., Georges D., Bray F. (2020). Global burden of cancer attributable to infections in 2018: a worldwide incidence analysis. Lancet Global Health.

[19] Hosaka T., Suzuki F., Kobayashi M. (2013). Long-term entecavir treatment reduces hepatocellular carcinoma incidence in patients with hepatitis B virus infection. Hepatology.

[20] Inoue J., Minami S., Abe K. (2025). Validation study of scores predicting hepatocellular carcinoma risk in chronic hepatitis B patients treated with nucleos(t)ide analogues. J Viral Hepat.

[21] Na J.E., Sinn D.H., Lee J.H. (2021). Efficacy of entecavir versus tenofovir in preventing hepatocellular carcinoma in patients with chronic hepatitis B with maintained virologic response. J Viral Hepat.

[22] Kim S.U., Seo Y.S., Lee H.A. (2019). A multicenter study of entecavir *vs*. tenofovir on prognosis of treatment-naïve chronic hepatitis B in South Korea. J Hepatol.

[23] Pinheiro P.S., Zhang J.J., Setiawan V.W. (2025). Liver cancer etiology in Asian subgroups and American Indian, black, Latino, and white populations. JAMA Netw Open.

[24] Cao G.Y., Liu J., Liu M. (2022). Trends in mortality of liver disease due to hepatitis B in China from 1990 to 2019: findings from the global burden of disease study. Chin Med J.

[25] Qiu S.K., Cai J.Y., Yang Z.P. (2024). Trends in hepatocellular carcinoma mortality rates in the US and projections through 2040. JAMA Netw Open.

[26] Li C.L., Hsu C.L., Lin Y.Y. (2024). HBV DNA integration into telomerase or MLL4 genes and TERT promoter point mutation as three independent signatures in subgrouping HBV-related HCC with distinct features. Liver Cancer.

[27] Péneau C., Imbeaud S., la Bella T. (2022). Hepatitis B virus integrations promote local and distant oncogenic driver alterations in hepatocellular carcinoma. Gut.

[28] Qian Z.Y., Liang J.B., Huang R. (2024). HBV integrations reshaping genomic structures promote hepatocellular carcinoma. Gut.

[29] Li W.Y., Cui X.F., Huo Q. (2019). Profile of HBV integration in the plasma DNA of hepatocellular carcinoma patients. Curr Genom.

[30] Yang X.B., Wu L.C., Lin J.Z. (2017). Distinct hepatitis B virus integration patterns in hepatocellular carcinoma and adjacent normal liver tissue. Int J Cancer.

[31] Yang L., Ye S., Zhao X.Y. (2018). Molecular characterization of HBV DNA integration in patients with hepatitis and hepatocellular carcinoma. J Cancer.

[32] Kim J.S., Kim H.S., Tak K.Y. (2025). Male preference for TERT alterations and HBV integration in young-age HBV-related HCC: implications for sex disparity. Clin Mol Hepatol.

[33] Jang J.W., Kim H.S., Kim J.S. (2021). Distinct patterns of HBV integration and *TERT* alterations between in tumor and non-tumor tissue in patients with hepatocellular carcinoma. Int J Mol Sci.

[34] Hui R.W., Wong D.K., Lyu X.Y. (2025). Profiles of HBV DNA integration in humans with hepatitis B virus infection: insights for antiviral treatment. JHEP Rep.

[35] Wen X.J., Wu X.N., Sun Y.M. (2024). Long-term antiviral therapy is associated with changes in the profile of transcriptionally active HBV integration in the livers of patients with CHB. J Med Virol.

[36] Yu X.Q., Gong Q.M., Yu D.M. (2024). Spatial transcriptomics reveals a low extent of transcriptionally active hepatitis B virus integration in patients with HBsAg loss. Gut.

[37] Gao N., Guan G.W., Xu G.L. (2023). Integrated HBV DNA and cccDNA maintain transcriptional activity in intrahepatic HBsAg-positive patients with functional cure following PEG-IFN-based therapy. Aliment Pharmacol Ther.

[38] Wang H., Hu B.B., Liang H.K. (2024). Impact of HBV integration on hepatocellular carcinoma after long-term antiviral therapy. Int J Gen Med.

[39] Song J., Zhang X.C., Ge Q.Y. (2018). CRISPR/Cas9-mediated knockout of HBsAg inhibits proliferation and tumorigenicity of HBV-positive hepatocellular carcinoma cells. J Cell Biochem.

[40] Jing X., Tian Z.B., Gao P.J. (2018). HBsAg/β2GPI activates the NF-κB pathway *via* the TLR4/MyD88/IκBα axis in hepatocellular carcinoma. Oncol Rep.

[41] Ahluwalia S., Ahmad B., Salim U. (2022). Hepatitis B virus-encoded HBsAg contributes to hepatocarcinogenesis by inducing the oncogenic long noncoding RNA LINC00665 through the NF-κB pathway. Microbiol Spectr.

[42] Qin Y.F., Zhou Z.Y., Fu H.W. (2023). Hepatitis B virus surface antigen promotes stemness of hepatocellular carcinoma through regulating microRNA-203a. J Clin Transl Hepatol.

[43] Sivasudhan E., Blake N., Lu Z.L. (2022). Hepatitis B viral protein HBx and the molecular mechanisms modulating the hallmarks of hepatocellular carcinoma: a comprehensive review. Cells.

[44] Zhang Y., Cobleigh M.A., Lian J.Q. (2011). A proinflammatory role for interleukin-22 in the immune response to hepatitis B virus. Gastroenterology.

[45] Chen L.J., Lin X.Y., Lei Y.M. (2022). Aerobic glycolysis enhances HBx-initiated hepatocellular carcinogenesis *via* NF-κBp65/HK2 signalling. J Exp Clin Cancer Res.

[46] Yuan H.M., Xu R.C., Li S.L. (2025). The malignant transformation of viral hepatitis to hepatocellular carcinoma: mechanisms and interventions. MedComm.

[47] Liu K., Chen E.B., Liang J.M. (2025). Single-cell analysis reveals distinct immune characteristics of hepatocellular carcinoma in HBV-positive *vs*. HBV-negative cases. Mol Clin Oncol.

[48] Lee S.K., Lim J., Jhun J.Y. (2025). Landscape of T-cell exhaustion heterogeneity and HBV integration in virus-related HCC revealed by whole-exome, transcriptome, and single-cell sequencing. JHEP Rep.

[49] Liu L.L., Liu J.W., Li P. (2023). Single-cell analysis reveals HBV-specific PD-1^+^CD8^+^ TRM cells in tumor borders are associated with HBV-related hepatic damage and fibrosis in HCC patients. J Exp Clin Cancer Res.

[50] Kaneko S., Kurosaki M., Joko K. (2020). Detectable HBV DNA during nucleos(t)ide analogues stratifies predictive hepatocellular carcinoma risk score. Sci Rep.

[51] Kim G.A., Lim Y.S., Han S. (2024). Viral load-based prediction of hepatocellular carcinoma risk in noncirrhotic patients with chronic hepatitis B: a multinational study for the development and external validation of a new prognostic model. Ann Intern Med.

[52] Choi W.M., Yip T.C., Kim W.R. (2024). Chronic hepatitis B baseline viral load and on-treatment liver cancer risk: a multinational cohort study of HBeAg-positive patients. Hepatology.

[53] Raffetti E., Fattovich G., Donato F. (2016). Incidence of hepatocellular carcinoma in untreated subjects with chronic hepatitis B: a systematic review and meta-analysis. Liver Int.

[54] Lyu X., Liu K., Chen Y.D. (2016). Analysis of risk factors associated with the development of hepatocellular carcinoma in chronic HBV-infected Chinese: a meta-analysis. Int J Environ Res Publ Health.

[55] Li Z.L., Han J., Liu K. (2019). Association of family history with long-term prognosis in patients undergoing liver resection of HBV-related hepatocellular carcinoma. Hepatobiliary Surg Nutr.

[56] Chatsirisupachai K., Lesluyes T., Paraoan L. (2021). An integrative analysis of the age-associated multi-omic landscape across cancers. Nat Commun.

[57] Zhao J.Y., Fang L.T., Pu R. (2024). Androgen receptor-induced molecules and androgen contribute synergistically to male-predominance of hepatocellular carcinoma. iScience.

[58] Xu C.G., Cheng S.Y., Chen K. (2023). Sex differences in genomic features of hepatitis B-associated hepatocellular carcinoma with distinct antitumor immunity. Cell Mol Gastroenterol Hepatol.

[59] Ringelhan M., Schuehle S., van de Klundert M. (2024). HBV-related HCC development in mice is STAT3 dependent and indicates an oncogenic effect of HBx. JHEP Rep.

[60] Chen C.J., Yang H.I. (2011). Natural history of chronic hepatitis B REVEALed. J Gastroenterol Hepatol.

[61] McMahon B.J., Nolen L.D., Snowball M. (2021). HBV genotype: a significant risk factor in determining which patients with chronic HBV infection should undergo surveillance for HCC: the hepatitis B Alaska study. Hepatology.

[62] Nishida N., Kudo M. (2013). Oxidative stress and epigenetic instability in human hepatocarcinogenesis. Dig Dis.

[63] Qiao K.Y., Zhang S.T., Trieu C. (2017). Genetic polymorphism of MTHFR C677T influences susceptibility to HBV-related hepatocellular carcinoma in a Chinese population: a case-control study. Clin Lab.

[64] Sun Z., Lu P., Gail M.H. (1999). Increased risk of hepatocellular carcinoma in male hepatitis B surface antigen carriers with chronic hepatitis who have detectable urinary aflatoxin metabolite M1. Hepatology.

[65] Kelleher T.B., Afdhal N. (2006). Assessment of liver fibrosis in co-infected patients. J Hepatol.

[66] Zhou H.X., Ba J.H., Xiao C.Y. (2026). Alcohol activates ATF4/LPLA2-mediated BMP metabolism to enhance HBV-induced hepatocellular carcinogenesis. J Hepatol.

[67] Wang J., Qiu K.J., Zhou S.S. (2025). Risk factors for hepatocellular carcinoma: an umbrella review of systematic review and meta-analysis. Ann Med.

[68] Harford K.A., Reynolds C.M., McGillicuddy F.C. (2011). Fats, inflammation and insulin resistance: insights to the role of macrophage and T-cell accumulation in adipose tissue. Proc Nutr Soc.

[69] Jin J., Kouznetsova V.L., Kesari S. (2023). Synergism in actions of HBV with aflatoxin in cancer development. Toxicology.

[70] Sou F.M., Hu T.H., Hung C.H. (2020). Incidence and predictors of hepatocellular carcinoma beyond year 5 of entecavir therapy in chronic hepatitis B patients. Hepatol Int.

[71] Wu C.Y., Lin J.T., Ho H.J. (2014). Association of nucleos(t)ide analogue therapy with reduced risk of hepatocellular carcinoma in patients with chronic hepatitis B: a nationwide cohort study. Gastroenterology.

[72] Cho J.Y., Paik Y.H., Sohn W. (2014). Patients with chronic hepatitis B treated with oral antiviral therapy retain a higher risk for HCC compared with patients with inactive stage disease. Gut.

[73] Hsu Y.C., Wong G.L., Chen C.H. (2020). Tenofovir versus entecavir for hepatocellular carcinoma prevention in an international consortium of chronic hepatitis B. Am J Gastroenterol.

[74] Kaneko S., Kurosaki M., Mashiba T. (2023). Risk factors for hepatocellular carcinoma at baseline and 1 year after initiation of nucleos(t)ide analog therapy for chronic hepatitis B. J Med Virol.

[75] Keitoku T., Tamaki N., Kurosaki M. (2023). Effect of fatty liver and fibrosis on hepatocellular carcinoma development in patients with chronic hepatitis B who received nucleic acid analog therapy. J Viral Hepat.

[76] Lin S.M., Sheen I.S., Chien R.N. (1999). Long-term beneficial effect of interferon therapy in patients with chronic hepatitis B virus infection. Hepatology.

[77] Papatheodoridis G.V., Manesis E., Hadziyannis S.J. (2001). The long-term outcome of interferon-alpha treated and untreated patients with HBeAg-negative chronic hepatitis B. J Hepatol.

[78] Lin S.M., Yu M.L., Lee C.M. (2007). Interferon therapy in HBeAg positive chronic hepatitis reduces progression to cirrhosis and hepatocellular carcinoma. J Hepatol.

[79] Lee D., Chung Y.H., Lee S.H. (2012). Effect of response to interferon-α therapy on the occurrence of hepatocellular carcinoma in patients with chronic hepatitis B. Dig Dis.

[80] Liang K.H., Hsu C.W., Chang M.L. (2016). Peginterferon is superior to nucleos(t)ide analogues for prevention of hepatocellular carcinoma in chronic hepatitis B. J Infect Dis.

[81] Li S.Y., Li H., Xiong Y.L. (2017). Peginterferon is preferable to entecavir for prevention of unfavourable events in patients with HBeAg-positive chronic hepatitis B: a five-year observational cohort study. J Viral Hepat.

[82] Ren P.P., Cao Z.J., Mo R.D. (2018). Interferon-based treatment is superior to nucleos(t)ide analog in reducing HBV-related hepatocellular carcinoma for chronic hepatitis B patients at high risk. Expert Opin Biol Ther.

[83] Cheng K.L., Chen Y., Wang X.M. (2022). Entecavir combined with interferon-α is superior to entecavir monotherapy in reducing hepatic and extrahepatic cancer in patients with chronic hepatitis B. Cancer.

[84] Mao Q.G., Liang H.Q., Yin Y.L. (2022). Comparison of Interferon-α-based therapy and nucleos(t)ide analogs in preventing adverse outcomes in patients with chronic hepatitis B. Clin Res Hepatol Gastroenterol.

[85] Jiang S.W., Guo S.M., Huang Y. (2024). Interim analysis of the PARADISE study: benefits of add-on peginterferon-α in NA-treated patients with CHB. Antivir Res.

[86] Chow N., Wong D., Lai C.L. (2023). Effect of antiviral treatment on hepatitis B virus integration and hepatocyte clonal expansion. Clin Infect Dis.

[87] Wang G., Hou F., Xie Q. (2024). Peginterferon alpha-2b combined with TDF promotes durable HBsAg loss in patients with chronic hepatitis B: a multicenter, randomized controlled, phase 3 clinical trial. Hepatology.

[88] Bruder Costa J., Dufeu-Duchesne T., Leroy V. (2016). Pegylated interferon α-2a triggers NK-cell functionality and specific T-cell responses in patients with chronic HBV infection without HBsAg seroconversion. PLoS One.

[89] Islam M., Kumar K., Sevak J.K. (2023). Immune drivers of HBsAg loss in HBeAg-negative CHB patients after stopping nucleotide analog and administration of Peg-IFN. Hepatol Commun.

[90] Wang D.Y., Fu B.Q., Shen X.K. (2021). Restoration of HBV-specific CD8^+^ T-cell responses by sequential low-dose IL-2 treatment in non-responder patients after IFN-α therapy. Signal Transduct Targeted Ther.

[91] Kim G.A., Lee H.C., Kim M.J. (2015). Incidence of hepatocellular carcinoma after HBsAg seroclearance in chronic hepatitis B patients: a need for surveillance. J Hepatol.

[92] Gounder P.P., Bulkow L.R., Snowball M. (2016). Nested case-control study: hepatocellular carcinoma risk after hepatitis B surface antigen seroclearance. Aliment Pharmacol Ther.

[93] Yip T.C., Chan H.L., Wong V.W. (2017). Impact of age and gender on risk of hepatocellular carcinoma after hepatitis B surface antigen seroclearance. J Hepatol.

[94] Yip T.C., Wong G.L., Chan H.L. (2019). HBsAg seroclearance further reduces hepatocellular carcinoma risk after complete viral suppression with nucleos(t)ide analogues. J Hepatol.

[95] Yip T.C., Wong V.W., Tse Y.K. (2021). Similarly low risk of hepatocellular carcinoma after either spontaneous or nucleos(t)ide analogue-induced hepatitis B surface antigen loss. Aliment Pharmacol Ther.

[96] Yip T.C., Wong V.W., Lai M.S. (2023). Risk of hepatic decompensation but not hepatocellular carcinoma decreases over time in patients with hepatitis B surface antigen loss. J Hepatol.

[97] Liu F., Wang X.W., Chen L. (2016). Systematic review with meta-analysis: development of hepatocellular carcinoma in chronic hepatitis B patients with hepatitis B surface antigen seroclearance. Aliment Pharmacol Ther.

[98] Anderson R.T., Choi H.S.J., Lenz O. (2021). Association between seroclearance of hepatitis B surface antigen and long-term clinical outcomes of patients with chronic hepatitis B virus infection: systematic review and meta-analysis. Clin Gastroenterol Hepatol.

[99] Song A.X., Wang X.X., Lu J.F. (2021). Durability of hepatitis B surface antigen seroclearance and subsequent risk for hepatocellular carcinoma: a meta-analysis. J Viral Hepat.

[100] Vittal A., Sharma D., Hu A. (2022). Systematic review with meta-analysis: the impact of functional cure on clinical outcomes in patients with chronic hepatitis B. Aliment Pharmacol Ther.

[101] Wu D., Kao J.H., Piratvisuth T. (2025). Update on the treatment navigation for functional cure of chronic hepatitis B: expert consensus 2.0. Clin Mol Hepatol.

[102] Author Group of Expert Consensus (2025). Chinese expert consensus on clinical practice of chronic hepatitis B functional (clinical) cure. Chin J Hepatol.

